# Detecting Glutathione and Related Antioxidants as Biomarkers in Patient Breast Tumor Tissues: An Update in the Age of Metabolomics

**DOI:** 10.1155/omcl/1811206

**Published:** 2025-12-10

**Authors:** Michael P. Gamcsik

**Affiliations:** ^1^ Lampe UNC/NCSU Joint Department of Biomedical Engineering, North Carolina State University, Raleigh, North Carolina, USA, ncsu.edu

**Keywords:** ascorbate, biomarker, breast cancer, glutathione, patients, taurine

## Abstract

Increased levels of glutathione (GSH) and related antioxidant processes are thought to predict breast tumor aggressiveness and therapy response. In our 2012 review of 21 studies, we found that most patient breast tumors exhibited increased GSH levels compared to peritumoral tissue. However, there was no clear relationship between GSH levels and histological grade, clinical stage, or patient outcome. For this update, database searches found 59 studies that reported the levels of any of 10 metabolites, including GSH, cysteine (Cys), ascorbate (Asc), and taurine (Tau), in breast tumor tissues. The increase in the number of studies profiling tumor metabolites is mainly due to the use of an array of relatively new metabolomics technologies. However, many of these metabolomics methods are not designed to prevent sample oxidation during tissue procurement and processing. Despite this, these recent studies confirm that the levels of most of the antioxidants or related metabolites are increased in patient breast tumor tissues compared to normal tissues. In addition, poor patient outcomes are often associated with tumor tissues with higher GSH and lower Tau levels. GSH levels also increase with histological grade. There are no clear trends in the relationship between any of the antioxidant levels and tumor stage or genetically defined subtypes. Clearer trends may emerge with more uniform tissue sampling, preparation, and assay procedures. In addition, the increased use of spatial metabolomics methods may help to clarify the relationship between antioxidant levels and clinical markers.

## 1. Introduction

All proliferating tissues, whether healthy or cancerous, display a highly reduced intracellular environment, which is reflected in the high reduced‐to‐oxidized (redox) ratio of the levels of glutathione (GSH) to its oxidized disulfide (GSSG) [1, 2]. These observations suggest that altered GSH metabolism is a common marker of cancer. A reduction in this ratio, for example, caused by an increasingly oxidative environment, can lead to cellular differentiation and ultimately cell death [1–3]. Many cancer therapies directly or indirectly alter the redox balance by inducing oxidative stress, which results in cell death [4–6]. It has long been hypothesized that the resistance of cancer cells to these therapies can be linked to increased levels of antioxidant proteins and small molecules [7, 8]. We summarized many of these studies in 2012 and found that most human solid tumors do indeed display altered GSH metabolism relative to healthy tissues [9]. However, the relationship between GSH levels, tumor aggressiveness, and patient outcomes was not clear from the data presented in those studies. Since that review, the analytical methods and technologies that produce much of the clinical data on GSH levels have changed. The more recent molecular biomarker screenings of patient tumors utilize one or several metabolomics technologies. Based on a PubMed search, the number of published metabolomic studies has increased by almost an order of magnitude since 2012, and these untargeted studies often capture data on other antioxidants and related metabolites in addition to GSH. However, the problem with many of these broad molecular surveys is that they are untargeted and not tailored to preserve antioxidant levels, as they are susceptible to oxidation or degradation during sample preparation or analytical processes. In our previous review, many of the summarized GSH studies utilized targeted assays and often took precautions to best preserve the molecular integrity or acknowledged that the assays measure “total glutathione,” representing the amount of both reduced, GSH, and GSSG species, or included other low‐molecular‐weight thiols [9]. Many untargeted metabolomics technologies rely on nuclear magnetic resonance (NMR) spectroscopy or mass spectrometry (MS) to obtain metabolite profiles, and we summarize the advantages and shortcomings of each method in relation to assessing antioxidant content. In addition, MS imaging (MSI) has been increasingly used to analyze patient tumors and offers significant advantages in molecular screening. The explosion in metabolomics data from all clinical tumor studies cannot be summarized in a single review; therefore, as a follow‐up to our 2012 review, this communication focuses on GSH and other related biomarkers found in patient breast tumor tissues.

## 2. Methods

### 2.1. Data Search

PubMed and Web of Science databases were used to identify studies published between 2012 and August 2025. The following keyword combinations were used to identify eligible publications.

• Human AND breast AND cancer AND metabolomics AND patients NOT serum NOT plasma.
• Glutathione AND human AND patient AND tissue AND antioxidant AND breast NOT serum NOT plasma NOT blood.
• Antioxidants AND breast AND cancer AND patient AND tissue AND metabolite NOT serum NOT plasma NOT blood.

### 2.2. Screening Process

Studies reporting the levels of these metabolites in plasma, serum, other blood components, urine, saliva, or feces were excluded. Studies reporting only metabolic pathway‐associated genes or proteins and no low‐molecular‐weight metabolites were excluded. Metabolomic studies utilizing patient‐derived samples and cultured in monolayers or organoids or implanted in mice as xenografts were excluded.

### 2.3. Inclusion Criteria

All studies that qualified for inclusion in this review reported original data on the absolute or relative levels of at least one of 10 metabolites: GSH, GSSG, cysteine (Cys), cystine (Cys2), cysteinyl‐glycine (CG), γ‐glutamyl‐Cys (GC), Cys‐glutathione disulfide (CSSG), ascorbate (Asc), dehydroascorbate (DHA), taurine (Tau) in patient breast tumor tissue.

## 3. Results

**Figure 1:**
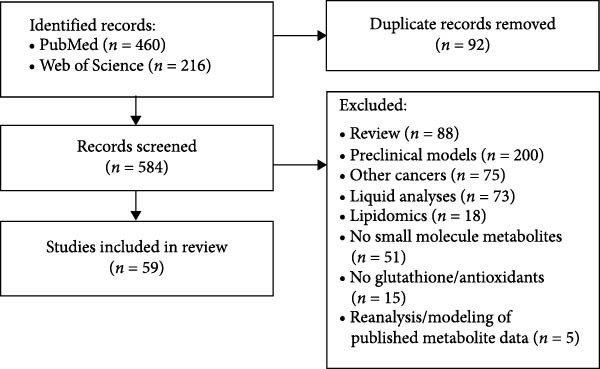
Flowchart of the study selection process.

Using the three keyword search combinations listed in the Methods section, PubMed returned 460 results, and Web of Science returned 216 (Figure 1). After removing duplicate citations, we obtained 584 unique results. Abstracts, full papers, and supporting information were searched; 520 publications were considered irrelevant. Most of the 520 studies involved preclinical models, either in vitro or in vivo rodent systems using established cell lines or patient‐derived tissues. Relevant articles were often found only upon examination of figures and supporting information, as common search functions using the 10 metabolites as keywords often scan only the main text. A total of 59 publications that included one or more of the 10 metabolites were identified. Five studies used previously published metabolite data from one or more of the 59 publications for reanalysis/model construction or used preclinical models to detect unique metabolite signatures in the published patient data.

Compared to the studies covered in the previous review in 2012, which often used GSH‐targeted assays, most of the studies covered herein are untargeted assays using different metabolomic technologies. Both targeted and untargeted assays are dependent on proper sample selection, acquisition, and processing procedures. Many metabolites listed in this review can be lost or degraded during these procedures. Therefore, prior to the review of patient data, the strengths and weaknesses of tissue collection and processing procedures and the metabolomic technologies widely used today are summarized.

### 3.1. Tissue Sample Selection and Acquisition

In metabolomics studies, biopsy specimens of breast tumor tissue are used for both histological and molecular analyses, and many studies have compared the molecular profiles of tumor stages, grades, types, and subtypes to determine whether metabolite biomarkers can improve tissue classification. Other studies compared tumor metabolite profiles to tissue from healthy controls (HCs); however, the definition of “healthy controls” varies, as this can be from tissue obtained from healthy patients undergoing reduction mammoplasties [10–12], or most commonly, from histologically normal‐appearing peritumoral breast tissue. This latter practice is most convenient, as paired tumor and peritumoral tissue can be acquired during the same procedure, thereby reducing inter‐patient variability and increasing the number of samples available for comparison. However, Chen et al. [13] compared GSH levels in ductal carcinoma in situ (DCIS), invasive ductal carcinoma (IDC), peritumoral adjacent benign tissue (ABT), and distant (DST) tissue defined as >5 cm from the tumor using MSI. DCIS and IDC exhibited similar GSH levels, which were significantly higher than DST levels. GSH levels in ABT tissues were also higher than those in DST tissues. The influence of a tumor on adjacent histologically normal‐appearing ABT, known as field cancerization, can alter gene, transcript, and metabolite content, but has not been widely reported in relation to GSH or other antioxidant levels [14]. In addition to the study by Chen et al., study, Gong et al. [15] sampled normal‐appearing tissue at >2 cm and Skorupa et al. [16] obtained multiple samples at the tumor border and at distances <1 cm, = 1 cm, and >1 cm DST from the tumor, however, in most studies, the proximity of the ABT sample site to the tumor is not reported (NR). Therefore, most studies compare tumor tissue biomarkers to peritumoral tissue, which appears to be histologically normal but it is not molecularly “normal” and this must be acknowledged when evaluating molecular profiles. However, if the two studies were designed to delineate tumor margins [17, 18], then a comparison of tumor and ABT profiles is warranted.

In addition to identifying control tissues, the requirements for tissue acquisition and handling vary between analytical methods (see below), and because of the well‐known molecular heterogeneity of tumors, single small biopsy samples may not represent the variation in metabolites across the tumor. Many studies include pathological examination of the cellular content of the tissue to ensure that an adequate fraction of tumor cells is present in each biopsy specimen or, for normal tissue, a high fraction of epithelial cells. This appears to be a reasonable precaution, but Choi et al. [19] obtained multiple tissue samples from tumors and documented the heterogeneity in cellular types (tumor cells, stroma, fibroblasts) and concluded that the intratumoral differences in cell types do not significantly correlate with differences in metabolite levels. Interestingly, tissue adipocytes, which are often excluded from analysis, were found to produce GSH and promote tumor progression in mouse models [20], suggesting that this tissue should also be included in assessing overall GSH content in human tumors. With the inherent heterogeneity of tumors, as expected, Gogiashivili et al. [21] detected a high variation in GSH levels within the same tumor; however, these differences were lower than those between tumors from different patients. Park et al. [22] compared specimens obtained from surgical resections to those from needle biopsies obtained from the central regions and peripheries of a mixture of breast tumor tissue subtypes and analyzed molecular profiles using a solid‐state NMR method termed high‐resolution magic angle spinning (HRMAS). The only antioxidant‐related metabolite reported in this study was Tau, and that, along with most other metabolites, was not statistically different between the sampling method used or location in the tumor center or periphery. Yang et al. [23] obtained punch biopsy samples of tumor central and peripheral tissues from luminal breast cancer and found spatial differences in GSSG and Tau levels in a solution‐state NMR‐based metabolomics study.

Once samples were procured, most studies indicated that the tissues were frozen and stored at −80°C prior to analysis. Many studies used terms such as “fresh frozen” or “immediately frozen” to describe how quickly the biopsy specimens are treated to reduce sample degradation, as the time interval between tissue acquisition and freezing may affect the levels of labile metabolites. For example, Haukaas et al. [24] determined the effect of various sample handling methods on metabolite content. They found that freezing alone can lower the levels of Asc and GSH, but the changes in Tau were not statistically significant. It is impractical to obtain and process multiple patient tissues in different clinical settings without freezing the sample. However, delaying freezing resulted in a steady reduction in the levels of Asc and GSH, which reached statistical significance at 60 and 90 min, respectively. Paradoxically, in a study on human liver tissue, Goosens et al. [25] showed that freezing delays of 120 and 150 min increased GSH levels without changing GSSG levels. Unfortunately, most studies do not report the interval between tissue acquisition and freezing, except for three, with periods of <1 min [26], <15 min [27] or <30 min [28], and it is likely that in other studies, these periods can be quite variable even within the same patient cohort. A possible alternative is to vacuum seal the sample for storage at 4°C [29]. This was shown to preserve Tau levels in breast cancer tissue for 72 h compared to flash‐frozen tissue, but choline, valine, and alanine levels changed.

For the effects of long‐term storage at −80°C, Madssen et al. [30] compared the molecular profiles of samples collected in two periods 1983–1989, 1994−1997 to samples banked between 2006 and 2009. The median RNA integrity number (RIN) indicated molecular degradation in the oldest banked samples. Multivariate statistical analyses revealed that metabolite profiles could discriminate between samples above or below the median RIN. Creatine‐ and choline‐containing species were the main metabolites that contributed to this difference. Although Tau was detected in the samples, it was not clear whether these levels differed among the older samples. Of the metabolites of interest in this review, Tau is likely the most stable to freeze–thaw cycles and long‐term storage [31]. In addition to freezing and storage variations, the preparation of samples for different analytical methods can potentially influence antioxidant stability.

### 3.2. Sample Preparation for Metabolomic Analysis

In our 2012 review of 21 studies on GSH in breast cancer patient tissues, most used spectrophotometric or chromatography‐based assays that were specific for GSH or non‐protein cellular sulfhydryls. However, the spectrophotometric assays often use a GSH reductase‐recycling approach that yields “total GSH” in the sample, which is assumed to be mainly the sum of GSH and GSSG content. Since then, most breast tissue studies have been untargeted metabolomic studies that use liquid chromatography–MS (LCMS), gas chromatography–MS (GCMS), and HRMAS. Several studies have combined analytical methods to obtain a comprehensive compendium of metabolites. Only one study used the targeted reductase‐recycling spectrophotometric method, which was used extensively in earlier work [32]. A list of analytical methods used to detect GSH or other antioxidants in the reviewed studies is presented in Table 1. Note that we may miss some relevant publications, as we found that GSH and other antioxidants were not mentioned in the main body of the study and only appeared in supplemental materials sections or in figures/diagrams, and therefore are not easily found in a keyword search.

**Table 1:** Analytical methods used in the reviewed studies.

Analytical method	No. of studies	Refs.
LCMS	27	[10–12, 15, 26–28, 33–52]
GCMS	12	[10, 11, 18, 27, 33, 37, 40, 51–55]
MSI	4	[13, 28, 56, 57]
CEMS	1	[58]
HRMAS	21	[16, 19, 21, 24, 30, 59–74]
NMR	2	[18, 23]
Spectrophotometric	1	[32]
IHC	1	[75]

*Note:* HRMAS, high‐resolution magic angle spinning magnetic resonance; NMR, solution‐state nuclear magnetic resonance spectroscopy.

Abbreviations: CEMS, capillary electrophoresis mass spectrometry; GCMS, gas chromatography–mass spectrometry; IHC, immunohistochemistry; LCMS, liquid chromatography–mass spectrometry; MSI, mass spectrometry imaging.

To analyze breast tissue GSH, each of the analytical methods listed in Table 1 requires different sample preparation steps that can impact the results. With all methods, it should be appreciated that GSH, Cys, and Asc are susceptible to oxidation. GSH and Cys can be oxidized to form many species, including homo‐ or mixed‐disulfides, along with mixed disulfides formed with Cys residues in proteins. GSH is also susceptible to enzymatic degradation by extracellular γ‐glutamyltranspeptidase (GGT) or intracellular γ‐glutamylcyclotransferase (ChaC1), which are often upregulated in tumor tissues [76].

For HRMAS, MSI, and IHC analyses, sample preparation is minimal; therefore, potential metabolite loss during processing may be reduced. Most biopsy tissues are frozen and stored. Tissue samples can remain frozen and sectioned, often using a cryostat at −20°C for MSI and IHC, which can slow sample oxidation or enzyme‐catalyzed changes. Only one study [13] reported the embedding medium used (optimal cutting temperature compound) and noted that this must be used sparingly, as it interferes with the ionization needed for MS analysis. Other embedding media may interfere with MSI detection or allow the diffusion of small metabolites such as GSH from the sample into the surrounding matrix [77]. Therefore, an optimized protocol for tissue embedding prior to sectioning for both MSI and IHC analyses was developed [77, 78]. MSI is a relatively new technique for metabolic screening that adds spatial information to analyses. For MSI, two studies in this review were performed using desorption electrospray ionization (DESI [13, 28]) MSI, and one using matrix‐assisted laser desorption ionization (MALDI [56]), which requires an extra step to coat the sample with an overlying matrix. For MALDI‐MSI, additional sample preparation steps may oxidize the sample [79]. Normally, samples for MSI analyses are maintained at room temperature and may take tens of minutes to collect. Samples for HRMAS do not require thin sections, and unprocessed tissue fragments of 5–10 mg are thawed and placed in the sample rotor, and analyses are often run at 5°C. HRMAS analyses usually take several minutes to complete. Leaving the samples at 5°C in the HRMAS rotor for 1.5 h was shown to result in changes in some metabolites, but both Asc and Tau were not significantly affected [24]. However, GSH in the minimally processed samples used for HRMAS analyses at 5°C may be susceptible to enzyme‐catalyzed degradation, but no study covered in this review investigated this possibility.

Sample preparation for LCMS or solution‐state NMR analyses requires additional steps. Often, this includes protein precipitation and metabolite extraction, which slows or prevents enzyme degradation. For LCMS, protein removal and metabolite dissolution often use water/organic solvent solutions. However, this step may also remove GSH metabolites [80], so care must be taken when selecting an appropriate system. Most of the studies reviewed here report that the solutions are then dried and rehydrated for analysis. However, the drying step may decrease GSH and increase GSSG, as shown in a study by Lu et al. [81]. The same report also showed that the levels of the reduced form of the coenzyme NADPH also diminished with sample drying, leading to increased levels of NADP^+^ [81]. This work suggests that other cellular antioxidants may also be sensitive to drying and recommends the best practices to follow in LC–GC–MS and NMR metabolomics studies. The most reliable LCMS method for reducing the artifactual oxidation of GSH to GSSG includes a thiol alkylation step [81, 82]. This step was NR in any of the studies outlined herein, likely because the alkylation reagents may interfere with the detection of other metabolites. GCMS often includes additional sample derivatization steps beyond LCMS, which can lead to analyte loss or oxidation.

Since sample collection, preparation, and analytical methods vary in these studies, both the reduced and corresponding oxidized forms of the metabolites of interest were included in this review and treated equally. For GSH, targeted studies in the past often reported “total glutathione” as GSH + GSSG; therefore, this approach is consistent with what has been reported previously. Notably, some of the reviewed studies reported only GSSG and not GSH. In normal tissues, GSH is often >10‐fold higher in concentration than GSSG [82], so studies that report only GSSG may indicate sample oxidation. However, there is not much data on the relative GSH:GSSG levels in patient tumor tissues, with reports showing this ratio >1 (e.g., [83]) and others <1 (e.g., [84]). Therefore, in this review, levels of reduced and oxidized pairs are grouped.

Although both methods are used extensively in patient tissue screening, the sensitivities of the HRMAS and LCMS methods are very different. HRMAS only detects analytes at the highest tissue concentrations, resulting in datasets with ~20 metabolites. Only three of the metabolites of interest are normally detected using this method: Tau, Asc, and GSH. The Tau signal is usually the strongest because of its high tissue concentration, followed by low‐intensity signals for Asc and GSH. Depending on the sample, the latter two metabolites were NR in many of the HRMAS‐based studies. Although limited in the number of detected metabolites, an advantage of HRMAS is that the detected signal can be easily related to tissue concentration. In contrast, LCMS can detect and identify hundreds of metabolites in a tissue sample. For example, 536 metabolites were detected using LCMS by Budhu et al. [33] of which 355 were identifiable and of sufficient abundance to be reliably measured. However, for any MS study, detection requires metabolite ionization, the extent of which can vary between metabolites. In spatial studies, the ionization of the same metabolite will vary across the tissue, which can be problematic when relating signal intensities to tissue concentrations.

For this review, published studies in which at least one of the following 10 metabolites was reported: Asc, DHA, GSH, GSSG, Cys, Cys2, GC, CG, GSH‐Cys mixed disulfide (CSSG), and Tau. Unlike GSH and Asc, Tau does not react rapidly with reactive oxygen species [85], but nonetheless it is a product of Cys metabolism and supports GSH metabolism and antioxidant processes [86]. Tau is detected in many HRMAS and MS studies reviewed herein and argues for inclusion in this review. The redox pair NAD(P)H and NAD(P)^+^ could also be included, but their levels are not widely reported in the reviewed studies, likely due to their instability [81, 87]. However, a 2018 study identified conditions amenable to preserving levels of these redox pairs in LCMS studies [88] and may result in more work evaluating these cofactors as biomarkers.

### 3.3. Tumor GSH and Antioxidant Levels Compared to Normal Tissue

The studies comparing GSH or other cellular antioxidants in patient breast tumor tissue to either peritumoral (peri) tissue or HCs are listed in Table 2. Most tumor samples were obtained from patients with clinical stage II tumors and had mixed histological grades.

**Table 2:** Glutathione and antioxidant levels in tumor versus normal tissues.

Cite	#BC	#HC	#Peri	Assay	Stage	Grade	GSH FC (BC vs. N)	GSSG FC (BC vs. N)	Other (BC vs. N)	Notes
[53]	271		98#	GCMS			~3	NR	↑­: Cys, Cys2, Tau NS: gCG, DHA	“Normal” tissue undefined Training and validation sets

[17]	228		43	HRMAS		1–26 2–100 3–80	NR	NR	↑­: Asc, Tau	Detecting tumor margins

[11]	31	6		LCMS, GCMS		1–2 2–7 3–20	d‐NR	d‐NR	↑­: Tau d‐NR: Asc, Cys, Cys2, DHA	

[33]	65		65	LCMS, GCMS	I–6 II–44 III–15	NA	2.6	NS	↑­: Asc, Cys, Cys2, Tau NS: CSSG	Unique profile for liver, pancreas, breast cancers

[46]	3		3	LCMS	—	—	5, 5, NS	15, 0.05, NS		Limited patient numbers

[10]	25	5		LCMS, GCMS	—	—	144, 274 (ER+, ER−)	36, 48 (ER+, ER−)	­↑: Asc, Cys, Cys2, CSSG, Tau	

[27]	137		65	LCMS, GCMS	I‐‐16 II‐‐91 III/IV‐‐30	1–9 2–30 3–78	d‐NR	>1	↑­: Cys, Cys2, Tau d‐NR: Asc	67 pts discovery; 70 pts validation

[36]	74		74	LCMS	I–27 II–41 III –6	1–37 2–15 3– 22	1.7	NS	NS: Cys, Cys2, gGC d‐NR: Tau	

[32]	27		27	Recycling	0 –5 I –4 II –7 III –8 IV–3	Low 9 Int 10 High 8	0.7	2.2		Serum and tumor profiles show same trends

[62]	67			HRMAS			d‐NR	NR	↑­: Tau	Malignant compared to benign tissues

[59]	82		47	HRMAS		1–3 2–10 3–25 U ‐‐9	>1		↑­: Tau	TNBC, LABC have different metabolomic profiles PA: Asc, Tau metabolism increased in AA patients

[56]	99		35	MSI	0–7 I–34 II–36 III–6 IV–1		10.6	10.2		

[52]	59		40	LCMS, GCMS					↑­: Cys2, Tau ↓: CG	PA: ↑ ­glutathione metabolism

[28]	68		55	MSI, LCMS	NA (DCIS)		>1	NR	↑­: Asc, Tau	

[47]	30		30	LCMS	I–11 II–12 III–6	1–4 2–10 3–15	>1	>1	↑­: Asc, Tau	PA: ↑­ glutathione, ascorbate/aldarate, cysteine/methionine metabolism

[18]	30		30	NMR, GCMS	I–5 II–17 III–8		NR	NR	↑­: Tau	PA: ↑­ glutathione

[12]	27	24		LCMS	I–10 II–10 III/IV–7		NR	NR	↑­: Tau	PA: ↑­ ascorbate/aldarate metabolism

[57]	40			MSI	I–9 II–29 III–5	1–17 2–20 3–6	NR	NR	↑­. Tau	All TNBC, also serum metabolomics data PA: Asc?

[35]	330		149	LCMS		2,3	d‐NR	>1	↑­: Cys ↓ Tau	All TNBC PA: ↑­ glutathione, ascorbate/aldarate, cysteine/methionine metabolism

[13]	241		80	MSI			~2	NR	↑­: Asc, Tau	GSH higher ABT vs. DST PA: ↑­ glutathione, taurine/hypotaurine metabolism

[16]	39		39	HRMAS	I–22 II–17	1–11 2–21 3‐‐6	NR	NR	↑­: Asc, Tau	Multiple peritumoral locations sampled

[45]	453		76	LCMS			NR	NR	↑­: Asc, Cys2, Tau	PA: ­↑ glutathione, ascorbate/aldarate, cysteine/methionine, taurine/hypotaurine metabolism; normal undefined

*Note: ↑*­, increased levels; ↓, decreased levels, #BC, number of breast cancer patients or tissue samples; #HC, number of healthy controls; #Peri, number of peritumoral samples. AA, African‐American patient cohort; Asc, Asc, Cys, Cys; Cys2, Cys2; CSSG, Cys‐GSH disulfide; d‐NR, detected but not reported; FC, fold‐change tumor/normal; GSSG, glutathione disulfide; NS, not significantly different.

Abbreviations: ABT, adjacent benign tissue; DST, distant surrounding tissue; gGC, g‐glutamyl‐cysteine; IDC, invasive ductal carcinoma; NR, not reported; PA, pathway analysis.

In some studies, changes in the levels of individual metabolites have not been reported, but the investigators performed pathway analyses (PAs) that analyzed trends in groups of metabolic pathway‐defined metabolite sets that aimed to associate metabolites with functional changes in tumor tissue. Often, this is based on metabolite‐centric data and a differential abundance (DA) statistical method, such as that used by Zhang et al. [34]. In studies that collected both metabolomic and proteomic data sets, changes in both metabolites and metabolic pathway‐associated proteins provide additional evidence for functional differences between cancerous tissue and normal tissue. For example, Xiao et al. [35] incorporated metabolomics with transcriptomic and genomic data in a comprehensive approach to create functional metabolic pathway models of triple‐negative breast cancer (TNBC), which can be used in therapeutic targeting and precision medicine. Although this review focuses on the levels of individual metabolites, univariate analyses of measured metabolite differences are not always reported, even if the results may be significantly different. Therefore, PAs that detect changes in groups of metabolites that may indicate the antioxidants of interest are altered in comparative studies are included.

Table 2 summarizes the 22 studies that collected molecular profiles comparing tumor tissue to breast tissue from healthy patients [10–12] or histologically normal‐appearing peritumoral tissue.

In some studies, GSH levels were NR (Table 2) in a list of metabolites that included other antioxidants or detected and NR (d‐NR), meaning it was not included as part of the statistical analysis presented in the article. As noted above, in HRMAS studies, only Tau may be reported, as the intensities of the Asc or GSH signals are too low for measurement. Other studies may only publish a list of the most significantly changed metabolites and relegate the rest to supplemental data files without statistical analysis and are included as d‐NR in this review. This most often occurs in LCMS or GCMS studies because this technology has the capability to detect, identify, and quantify hundreds of individual metabolites; therefore, statistical analyses of each may not be the focus of the work.

Thirteen studies (Table 2) reported and analyzed GSH and/or GSSG data, and 12/13 detected an average increase in breast cancer tissue compared to healthy or histologically normal tissues. Only one study reported a lower level of GSH [32], which was the only study using a targeted assay similar to most of the studies covered in our earlier review. However, these investigators observed an increase in oxidized GSSG.

The greatest increase of >100‐fold was reported in a study by Tang et al. [10], where comparisons were made between tumor tissue and tissue from healthy women undergoing mammoplasties rather than the majority of studies using peritumoral tissue. Whether this is consistently observed in similar control groups is unclear. Two other studies [11, 12] also used reduction mammoplasty tissue from healthy patients, but neither reported GSH or GSSG levels in their work. However, comparing Tau levels in these studies, Tang et al. [10] reported a >3‐fold increase in Tau levels in tumor tissue, which is similar to the findings of Smith et al. [12], whereas Brauer et al. [11] only detected a 1.2‐fold increase. The work of Chen et al. [13] showed that histologically normal‐appearing tissue located adjacent to the tumor had an ~20% higher GSH level compared to tissue located >5 cm away, which emphasizes the caution that must be used in comparing molecular profiles in tumor and peritumoral tissue. Another study [36] sampled peritumoral tissue “a sufficient distance” from the cancer and reported a significantly higher 1.7‐fold increase in mostly early‐stage, low‐grade tumor tissue. Skorupa et al. [16] found elevated levels of most metabolites, including Tau and Asc, in tumors compared to peritumoral tissue samples collected ≥1 cm from the tumor border, but GSH has not been reported in this HRMAS‐based study. The intratumoral fibrotic tissue also showed altered metabolite levels.

These results also highlight the advantage of adding spatial information to metabolomic data to evaluate the heterogeneity in molecular profiles. The LCMS and GCMS methods homogenize the tissues, and the HRMAS method samples the entire tissue specimen; therefore, all these approaches lose spatial information on metabolite distributions and average metabolite levels across the volume of tissue collected that may include multiple cell types, connective and necrotic tissues. The relatively recent introduction of MSI to provide spatial metabolomic data will improve our understanding of how the presence of a tumor alters nearby metabolism. Table 2 lists the three MSI‐based studies. Two studies [28, 57] reported metabolomic data from tissues with a spatial resolution of 200 μm, and one with a 50 μm resolution [56]. At these levels, metabolites can be determined in a single tissue section that may contain regions of DCIS, invasive breast cancer, and benign tissue [28], which facilitates the evaluation of intercellular influences on metabolism and the influence of localized microenvironments.

Several studies have focused on determining the metabolic characteristics that may be linked to the poorer outcomes observed in African‐American breast cancer patients. TNBC tissue from African‐American patients was compared to ER + tissue, and it was found that GSH, GSSG, and Cys were all elevated in TNBC compared to ER + tissues, but none reached statistical significance [37]. Terunuma et al. [27] in the discovery and validation of metabolomics datasets of breast cancer and adjacent normal tissue from European‐American and African‐American women, reported higher levels of GSSG, Cys2, CSSG, Cys, and Tau as part of the 50 metabolites showing the greatest increases in tumors compared to normal tissue. In another study, an HRMAS‐based metabolomics comparison of TNBC and luminal A breast cancer (LABC) subtypes in both African‐American and Caucasian women found that GSH and Tau levels were higher than those in adjacent peritumoral tissues in both subtypes and patient groups [59]. Only multivariate analyses of metabolic profiles differentiated between the two groups. In addition, PA showed 29 significantly altered metabolic pathways in African‐American women, including GSH, Asc/aldarate, Cys/methionine, and Tau/hypotaurine metabolism, compared to one altered pathway, pyrimidine metabolism, in Caucasian women. In a third study, breast cancer tissues from a group of non‐Hispanic black women were compared to non‐Hispanic white women, and higher Tau was found in cancer tissues, and PA also indicated higher Asc/aldarate metabolism in both groups [12]. These investigators also found a relationship between the breast microbiome and the tissue metabolome.

Table 2 also shows that 20 studies detected Tau, and 17 of these reports showed increased Tau levels in the cancer tissue; only one study showed a decrease [35], and another [36] in which this metabolite was detected, but no statistical data were reported. The next most frequently reported antioxidant was Asc and/or DHA, which was detected in 11 studies. Nine of 11 studies detected increased levels, two studies detected Asc but did not report statistics, and one [53] indicated a nonsignificant change. Although one study reported elevated levels of Tau in TNBC tissues, a schematic in this publication also indicated that Asc was elevated, but no data on this metabolite were presented [57].

Overall, these 22 studies provide additional confirmation that GSH and/or GSSG levels are elevated in tumors compared to normal tissues and are consistent with the results of our earlier review. In addition, most studies reported increased Tau and many increased Asc, confirming the general observation that metabolites related to maintaining a reduced cellular environment are altered in cancer tissues. The observation of increased Tau is consistent with results showing higher levels in a range of human tumor tissues, including breast cancer [89].

### 3.4. Tumor GSH and Antioxidant Levels in Relation to Clinical Markers

Many of the studies summarized above and additional studies listed in Table 3 also investigated whether the metabolomic profile could help stratify patient tumors already characterized by histologic grade, clinical stage, treatment response, or outcome. With a few studies collecting both genomic and transcriptomic data, investigators assessed whether metabolic profiles differentiate between hormone receptor status or align with the five molecular subtypes characterized by the PAM50 gene signature: luminal A (LumA), luminal B, human epidermal growth factor receptor 2 (HER2)‐enriched, basal‐like, and normal‐like.

**Table 3:** Glutathione and antioxidant levels in relation to clinical characteristics.

Cite	#Pts	Assay	Stage	Grade	ER (+/−)	PR (+/−)	Subtypes ‐‐ #Pts	Notes/results
[40]	186	GCMS, LCMS		1–20 2–128 3–78			Her2+–17	• ↑ ­ Tau and ↓ DHA associated with increased glycerol‐3‐phosphate acyltransferase (GPAM) expression that is linked to better overall survival. • NS correlation of Cys, Cys2, GSH with GPAM

[63]	34	HRMAS		1,2–24 3–10	26/6	14/18	Her2+–27 TNBC–4	• Examined profiles in relation to histologic prognostic factors • ↑ ­ Tau in PR−, Her2−, trended ↑ in ER−, poorer prognosis

[66]	98	HRMAS		1–8 2–50 3–39	71/24	60/34		• Metabolites related to 5‐year survival • Nonsurvivors: ↓Tau • d‐NR: Asc

[11]	31	LCMS, GCMS		1–2 2–7 3–20	20/10		Basal–10 Her2+–3 LumA–10 LumB–6 Normal–1	• Comparison between aggressive and nonaggressive tumors • Trend toward ­↑ Tau in more aggressive tumors • d‐NR: GSH, GSSG Asc, Cys, Cys

[55]	271	GCMS	I–86 II–238 III–27 IV–14	1–27 2–144 3–98	204/67	180/91	Her2+–31	• Metabolites in relation to ER+ vs. ER− • NS: GSH, Cys, Cys2, Cys‐gly, DHA, Tau

[69]	37	HRMAS	II–26 III–11		25/12	6/31	Her2+–11	• Predicting patient response to neoadjuvant chemotherapy • Tau levels not related to response

[33]		LCMS, GCMS	I–6 II–44 III–15					• Comparison of breast, liver, pancreatic tumor metabolites • ↑ ­ Tau in early‐stage breast cancer

[90]	75	HRMAS		1–6 2–22 3–30	44/31	32/43	Her2–30 TNBC–20 TPBC–11	• Metabolomic of TNBC • Tau was part of multivariate analyses that differentiate TNBC and TPBC, Her2+ from Her2− and hormone receptor status.

[37]	30	LCMS, GCMS	NR	Higher grade in TNBC	15/30		TNBC–15	• Metabolites in relation to ER+ vs. TNBC in AA patient • Elevated but not NS in GSH, GSSG; CSSG, Cys in TNBC • GSH or related metabolites elevated but NS with patient death.

[10]	25	LCMS, GCMS			14/9		LumA–10 LumB–5 Her2/neu+–2 Basal–5	• ↑ ­ GSH, ­↑ GSSG in ER− vs. ER+ • GSH, Asc positively correlated with BRCA1 mRNA • Asc, Cys, CSSG, positively correlated with Ki‐67 index

[27]	137	LCMS. GCMS	I–16 II–91 III/IV–30	1–9 2–30 3–78	33/104		TNBC–56	• Discovery /validation cohorts in search of biomarkers • ↑ ­ Tau, ↑­ GSSG, ­↑ Cys2, ↑­ Cys, ↑ CSSG in high grade • ↑­ GSH, ­↑ GSSG in ER+ vs. ER− tissues • PA: glutathione pathway elevated in poor prognosis group

[54]	274	GCMS	I–86 II–141 III–27 IV–14	1–27 2–145 3–99	204/66		Her2+–30	• Glutamate enriched tumor correlated with improved survival • Cys and Tau directly correlated with glutamate enrichment • Cys2, DHA, Cys‐gly show no correlation

[71]	60	HRMAS	DCIS/ DCIS(IDC)		40/20	32/28	Lum–40 Her2+–28	• Comparison of pure DCIS to DCIS with invasive component • NS: Tau between groups

[72]	228	HRMAS	DCIS–4 I–113 II–93 III–9	1–31 2–93 3–97	78/40	55/63	Her2+–26	• 3 metabolic clusters (MC1–MC3) not related to genetic subtypes • Asc differentiates MC2 from MC1,MC3 • GSH differentiates MC2 from MC3 • Tau differentiates MC1 from MC3

[67]	50	HRMAS					Indolent–6 Not indolent–44	• Metabolome of indolent breast cancer • NS: Tau in indolent tumors • ↓ Tau in recurrent tumors

[74]	53	HRMAS		1, 2–29 3–17	36/17	16/27		• Correlate metabolome to PET and/or MRI clinical parameters • Tau found to correlate only with MRI diffusion coefficient in Ki‐67 negative tumors

[51]	92	LCMS, GCMS					LumA–36 LumB–40 Her2–7 Basal–9	• Molecular profiles of subtypes • d‐NR: Asc

[73]	62	HRMAS					LumA–36 LumB–26 Her2+–10	• Metabolomic profiles in relation to clinical markers in ER‐positive tissues • ↑­ Tau in LumB compared to LumA and trended higher in Her2+ vs. Her2− tissues. • NS: Tau in Ki‐67 high compared to low.

[36]	74	LCMS		1–37 2–15 3–22			HR+–49 TNBC–11	• Metabolomics in TNBC vs. HR+ • NS: GSH, GSSG, Cys, Cys2 in TNBC vs. HR+ • ↑ ­ Cys in TNBC vs. normal tissue • NS: GSH, GSSG, Cys, Cys2 in HR+ vs. normal tissue

[32]	27	Spectro	0–5 I–4 II–7 III–8 IV–3	1–9 2–10 3–8				• GSH no correlation with Stage • GSSG no correlation with Stage

[59]	82	HRMAS		1–3 2–10 3–25			LumA–29 TNBC–18	• Compare African‐American (AA) to Caucasian BC patients • ↑ ­ GSH higher in AA TNBC vs. normal • All patients GSH, ↑­ Tau in both LumA and TNBC vs. normal • PA: glutathione, ascorbate/alderate, taurine/hypotaurine metabolism altered in AA patients

[58]	20	CEMS	0–5 I–5 II–4 III–1					• Profiles of benign, DCIS, IDC tissues • Cys, GSH, GSSG appear higher in IDC • PA: Cysteine/methionine metabolism higher in IDC

[30]	74	HRMAS	I–30 II–29 III–5 IV–6	1–8 2–28 3–24	49/25	40/30		• Validate metabolite markers using long‐term banked tissues • ↓ Asc in older sample • ­↑ Tau in ER+ and PR+ tissues

[41]	52	LCMS	I–21 II–24 III–7	1–5 2–22 3–24			Her2+–12 TNBC–15 Luminal–25	• Metabolomic data analyzed by 5 machine‐learning methods • Tissue from patients grouped in 3 prognosis clusters • GSH similar in favorable and unfavorable prognosis cluster and lower in intermediate prognosis cluster • PA: glutathione and ascorbate/aldarate metabolism can differentiate some clusters

[28]	68	MSI, LCMS	DCIS–5 DCIS(IBC)–10				LumA–13 LumB–17 Her2+–10 TNBC–12	• NS: GSH in DCIS vs. invasive BC or subtypes • NS: Tau in DCIS vs. invasive BC or subtypes • NS: Asc in DCIS vs. invasive BC, but was a factor in differentiating TNBC from other subtypes

[15]	72	LCMS					TNBC–72	• TNBC separated into 3 metabolic pathway based subtypes MPS1 – MPS3 with MPS2 poorest prognosis • GSSG different between all three groups • Tau, Cys2 different between 2 of 3 groups • GSH differentiates MPS2 vs. MPS3 • Cys‐gly differentiates MPS1 vs. MPS3 • PA: glutathione, cysteine/methionine, ascorbate/aldarate metabolism altered in MPS2 vs. MPS1 • Multivariate analyses ID therapeutic targets in MPS1, MPS2

[19]	46	HRMAS		1, 2–37 3–10	34/13	29/18	Her2+–9	• GSH not correlated with grade, ER, PR, HER2, Ki‐67 index, mets • ↑ ­ Tau in ER+: PR+ tumors, higher in low vs. high grade, not different in Ki‐67 nor Her2+: nor lymph node invasion.

[42]	24	LCMS			9/15		LumA–4 LumB–3 TNBC–15 Basal–9 Normal–7	• Comparison of TNBC to ER+ tissues • Cluster 1 (all healthy, all ER+, 7 TNBC), Cluster 2 (8/15) • ↑ ­ GSH, ↑­ Cys, ↑­ CG in Cluster 2 • PA; glutathione, ascorbate/aldarate, cysteine/methionine, taurine/hypotaurine enriched in cluster 2

[12]	27	LCMS	I–10 II–10 III/IV–7				LumA–12 TNBC–15	• Metabolome and microbiome of black and white patient samples • NS: Tau between Luminal and TNBC

[35]	330	LCMS					TNBC–330	• 3 metabolic clusters (C1–C3) identified in TNBC • Basal‐like tumors in subgroups C2, C3 with C2 worst RFS • PA: glutathione, ascorbate/aldarate metabolism enriched in C2 • GSSG negatively correlated with PIK3CA mutations and positively correlated with oncogene amplification

[44]	100	LCMS		1–10 2–42 3–48			Her2+–19	• Training and validation sets comparing Grade 3 to Grade 1,2 • ‘HomoGSH’ higher in Grade 3 vs. Grade 1,2 • PA: glutathione metabolism differ between Grades

[13]	241	MSI						• Profiles of DCIS, DCIS(IDC), IDC in tumor, ABT, DST • NS: GSH in DCIS(IDC) vs. DCIS • ↑­ Tau, Asc DCIS vs. IDC • PA: Ascorbate/aldarate metabolism differentiates low vs. high grade

[43]		LCMS					Her2−–91? Her2‐low– 434	• Study of molecular features of Her2‐low and BC • Three groups Her2+, Her2−, Her2‐low • PA: taurine/hypotaurine metabolism increased in HR− v HR+ Her2‐low BC • PA: NS: glutathione, cysteine/methionine, ascorbate/aldarate metabolism in HR− vs. HR+ Her2‐low

[49]	384	LCMS					All HR+/Her2−	• Molecular characterization of HR+/Her2− BC • Some correlations between Tau and Asc levels and oncogenic somatic mutations and copy number alterations

[16]	39	HRMAS		1–11 2–21 3–5	36/3	32/7	LumA–22 LumB–14 Her2+–1 TNBC–2	• ↑ ­ Tau, ↑ ­Asc increased with % tumor cells in higher‐grade tumors • ↑­ Asc in intratumor fibrosis in grade 1 tumors

[38]	59	LCMS					IMPC–25 IDC‐NOS–34	• Metabolomics of IMPC (poorer prognosis) and IDC‐NOS • Metabolic clusters differentiated IMPC (cluster 1), IDC‐NOS (cluster 2) and an “IMPC‐like” (cluster 3) intermediate state • PA: ascorbate/aldarate, taurine/hypotaurine metabolism higher in 3 vs. 2 • ↑ ­ GSH, ↑ ­ GGC, in Cluster 2 than Cluster 3 • ↑ ­ GSSG higher, Cys2 lower in Cluster 1 than Cluster 3

[48]	32	LCMS					Fibroadenoma–17 Phyllodes–15	• Metabolomics fibroadenomas vs. phyllodes (increased recurrence) BC • ↑ ­ GSSG found in phyllodes tissue • Also Raman spectroscopic evaluation

[39]	51	LCMS	I–9 II–21 III–18	1–1 2–41 3–2	38/13	38/13	LumA–10 LumB–28 Her2+–5 TNBC–8	• Comparison IMPC to IDC • GSSG lower in more invasive IMPC • PA: glutathione, cysteine/methionine downregulated in IMPC

[45]	453	LCMS					LumA–119 LumB–144 Her2+–98 Basal–52 Normal–35	• Multiomics study of Asian BC patients • Cys2, Tau lower in normal‐like • ↑­ Tau in LumB • ↓ Asc in Her2+ and basal‐like

[61]	228	HRMAS	I–49 II–130 III–147				LumA–79 LumB–72 Her2+–48 Basal–55 TNBC–46	• Multiomics training and validation cohorts for survival prediction • 3 Multiomics clusters (MOCs) ID with MOC2 poorest prognosis • Asc, GSH, Tau different between clusters; • PA: taurine/hypotaurine pathway differentiates MOC1, MOC2

[23]	18	NMR					LumA–10 LumB–8	• Multiomics study of central /peripheral tissues in LumA, B • ↑ ­ GSSG in periphery of LumA, LumB • ↑ ­ Tau in center of LumA, LumB

[50]	380	LCMS					All HR+/Her2−	• Multiomics‐based machine learning model to predict risk pre‐treatment samples • PA: taurine/hypotaurine pathway inversely correlated with risk score

*Note:* DCIS(IDC), ductal carcinoma in situ with invasive component; DST, normal tissue >5 cm from the tumor; FEC, 5‐fluorouracil/epirubicin/cyclophosphamide; GSSG, GSSGglutathione disulfide; HR, hormone receptor status, either ER or PR; LumA, luminal A subtype; LumB, luminal B subtype; NS, not significantly different.

Abbreviations: ABT, adjacent benign tissue; AC, adjuvant chemotherapy; Asc, ascorbate; BC, breast cancer; Bev, bevacizumab; Cys, cysteine; Cys2, cystine; CSSG, Cys‐glutathione disulfide; DCIS, ductal carcinoma in situ; DHA, dehydroascorbate; GGC, g‐glutamyl‐Cys; GSH, glutathione; IDC, invasive ductal carcinoma; IDC‐NOS, invasive ductal carcinoma not otherwise specified; IMPC, invasive micropapillary carcinoma; PA, pathway analysis; TNBC, triple‐negative breast cancer; TPBC, triple‐positive breast.

Table 3 presents the studies that collected metabolomic data, along with commonly collected clinical indicators. The notes/results column presents a brief description of the aim(s) of the study and whether any of the metabolites of interest are correlated with any of these clinical indicators.

In a separate category, a list of studies presenting metabolomic changes, patient responses, and outcomes induced by specific therapies is shown in Table 4. In these studies, treatments were variable and often NR. Additional details on the studies listed in Tables 3 and 4 are included in the following sections.

**Table 4:** Glutathione and antioxidant levels in treatment‐specific response.

Cite	#Pts	Assay	Stage	Grade	Er (+/−)	Pr (+/−)	Subtypes—#Pts	Treat Groups	Notes/Results
[64]	89	Hrmas	Ii– 28 Iii–50 Iv–5		41/19	32/28		Nac I–Epirubicin Ii–Paclitaxel	• Effect Of Nac On Metabolic Profile, Patient Response • Trend Toward Lower Tau In Nonsurvivors

[65]	33	Hrmas	Ii–5 Iii– 17 Iv ‐‐ 4	1 – 5 2 – 16 4 – 5	23/3	21/5		Nac– Doxorubicin	• Effect Of Nac On Metabolic Profile, Patient Response • Pretreatment Tau Was Best Predictor Of Response • Low Taurine:Glycine Ratios Was Found In Non‐Survivors

[75]	63	Ihc	I–4 Ii–48 Iii– 9 Iv– 2	1–3 2– 50 3– 10	43 /17	37/26	Luma–13 Lumb–22 Her2/Neu+ –41 Tnbc–9	I–Ac (Fec) Ii–Ac (Other) Iii–Surg	• Gsh Higher Er+ • Gsh Higher In Group Ii With Mets • Gpx Higher In Pr− • No Correlation Gsh/Gpx To Clinical Groups • No Correlation Gsh/Gpx To P54, Ki57

[60]	122	Hrmas			101/21	70/51		I–Nac (Fec) Ii–Nac + Bev	• Asc, Gsh. Tau Shows Changes In W/Chemotherapy • Gsh Show Additional Response To Bevacizumab

[26]	29	Lcms						Metformin	• Metabolic And Fdg‐Pet Response To Metformin • Gsh Trend Higher, Tau Trend Lower, Gssg No Change With Metformin • Pa: Glutathione, Cysteine/Methionine, Ascorbate/Aldarate Metabolism Increased

[68]	118	Hrmas			100/18	68/50		I–Ac (Fec) + Ctx + Bev Ii–Ac (Fec) + Ctx	• Ctx‐Induced Metabolic Differences Between Survivors And Non‐Survivors In Her2‐ Tissues • Tau, Asc Differentiated Between Survivors/Nonsurvivors

[70]	61	Hrmas	I–45 Ii– 16	1–11 2–30 3–20	50/11	41/20	Luma–39 Lumb–22	Dietary	• Effects Of Carbohydrate (Carb) Diet • Gsh Higher In Carb Vs. Fasting And In Er+ Vs. Er‐ • Gsh Correlated With High Serum Insulin Levels • High Gsh Content Increased Risk Of Relapse And Bc‐Specific Death

[34]	217	Lcms			85%/3%	69%/12%	Luma–31% Lumb–32% Normal–5% Basal–4% Her2+– 4%	Endocrine Ctx	• Multiomics Study Of Mainly Hr+/Her2− Patients Stratified By Response To Endocrine Ctx • Pa: Gsh, Ascorbate Higher In 1˚, 2˚, Recurrent Cancers

*Note:* EPH, ER+/PR+/Her2−; FEC, 5‐fluorouracil/epirubicin/cyclophosphamide; HR, hormone receptor status; LumA, luminal A subtype; LumB, luminal B subtype; CTx, chemotherapy.

Abbreviations: AC, adjuvant chemotherapy; BC, breast cancer; Bev, bevacizumab; IDC‐NOS, invasive ductal carcinoma not otherwise specified; IMPC, invasive micropapillary carcinoma; NAC, neoadjuvant chemotherapy; PA, pathway analysis; TNBC, triple‐negative breast cancer; TPBC, triple‐positive breast cancer.

#### 3.4.1. Patient Outcome

This outcome category was subdivided into GSH/GSSG and Tau levels related to patient prognosis/survival, as these two antioxidants were the most frequently reported.

##### 3.4.1.1. GSH and/or GSSG Data Are Reported Higher Poor Prognosis/Response/Survival in the Following 14 Studies

1. In a study comparing TNBC to ER+ breast cancer tissues from African‐American patients, the concentrations of GSH and Cys were elevated by 16‐ and 13‐fold, respectively, in tissues from non‐surviving patients; however, the difference was not statistically significant [37]. The TNBC tumors were of higher grade and poorly differentiated, and for these patients, the recurrence rate was higher, and the survival rate was lower (47% vs. 87%) compared to patients with ER+ tumors. Together with increases in other GSH‐related metabolites, the authors concluded that TNBCs have a “redox advantage” compared to the ER+ tumors.
2. Terunuma et al. [27] defined a subgroup of patient tissues that, based on DNA methylation patterns and MYC activation, was associated with poor prognosis. These features occur at a higher frequency in tissues of African‐American patients. PA revealed that the GSH metabolic pathway was 5‐fold higher in this subgroup.
3. Cluster analysis of transcriptomic data on TNBC tissue produced three metabolic subgroups: MPS1‐MPS2 [15]. Patients with tissues in the MPS2 subgroup had significantly worse relapse‐free survival (RFS) than those in other subgroups. Metabolomic data from 72 patients were used to validate these subgroups, and GSSG was increased in the MPS2 group and differed between the three subgroups. Tau was lower in MPS2.
4. In a study of 15 TNBC and 9 ER+ tissue samples, cluster analysis of the metabolomic data grouped all normal tissue, all ER+, and 7/15 TNBC samples as Cluster 1, and 8/15 TNBC tissues as Cluster 2. TNBC is more aggressive, and patients with these tumors have shorter survival times. Cluster 2, which featured only TNBC tissues, had higher GSH, Cys, and CG levels than Cluster 1. PA indicated that GSH, Cys/methionine, Asc/aldarate, and Tau/hypotaurine metabolisms were upregulated in cluster 2.
5. The same group collected metabolomic data from a larger patient population and confirmed the metabolic clustering of TNBC tissues into three subgroups [35]. In this study, GSSG was also the highest in the group with poor RFS, and PAs revealed that both GSH metabolism and Asc/aldarate metabolism pathways increased.
6. The metabolic profiles of invasive micropapillary carcinoma (IMPC) tissue were compared to IDC not otherwise specified (IDC‐NOS) and an intermediate state of “IMPC‐like tissue” in a study by Wu et al. [38]. Patients with IMPC tissues and poorer prognosis displayed higher GSH and GC levels than those in the other two groups. IMPC‐like tumors displayed increased GSSG levels compared to IDC‐NOS.
7. In patients with the HR+/HER2− breast tumor subtype who were treated with surgery and adjuvant endocrine therapy, delayed recurrence of the cancer is often observed. Zhang et al. [34] collected extensive multiomics data on this subtype and stratified patients into four groups: no relapse, primary resistant, secondary resistant, and endocrine sensitive. The latter three groups all showed recurrence at some point. PAs revealed that GSH metabolism and Asc/aldarate metabolism pathways were increased in all recurrent groups compared to the no‐relapse group. Tau/hypotaurine metabolism was lower in the primary resistant group but elevated in the secondary and endocrine sensitive groups compared to the no relapse group.

##### 3.4.1.2. GSH/GSSG Higher/Trended Higher in Good Prognosis/Response/Survival in Two Studies

8. In a study of HER2− breast cancer tissues, Euceda et al. [60] used HRMAS to track metabolite responses to chemotherapy alone or chemotherapy plus bevacizumab before treatment, 12 weeks into treatment, and at 24 weeks. Pre‐treatment, the tissue from patients in the good response (GR) group showed higher levels of GSH, Asc, and Tau compared to the no response (NR) group. At 24 weeks, all three levels were lower in the GR than in the NR group. These results may be considered a mixed response; however, the pre‐treatment data are most relevant if the aim is to use these metabolites in a predictive manner. In this study, GSH was the only metabolite altered by the addition of bevacizumab to the treatment regimen.
9. In a comparison of metabolite profiles of MPC, a highly invasive tumor, to IDC, GSSG levels were lower in IMPC [39]. PA revealed that GSH and Cys/methionine metabolism were downregulated in IMPC.

##### 3.4.1.3. GSH/GSSG: No Difference Related to Patient Prognosis/Response/Survival in Three Studies

10. Better overall survival of patients with tumor tissues expressing high levels of glycerol‐3‐phosphate acyltransferase (GPAM) was observed, but there was no significant difference in GSH levels between GPAM‐high and GPAM‐low tissues [40].
11. In a comparison of ER+ and ER− breast cancer tissues, Budczies et al. [54] found that a higher glutamate to glutamine ratio in ER− tumors correlated with tumor grade and overall survival. The GSH levels were not significantly different between the groups.
12. Five different machine learning methods were used to analyze a breast cancer metabolomic dataset, and 449 metabolites were clustered into three prognostic groups: C1, favorable; C2, intermediate; and C3, unfavorable [41]. Although GSH was identified as a key metabolite by four of the five analytical methods, its level was found to be lower in C2 than in the other groups but not significantly different between C1 and C3.

##### 3.4.1.4. GSH/GSSG Levels With Mixed Results in Two Studies

13. In the only IHC‐based study, investigators sampled three regions from each tumor section and 20 spots within each region to provide a good level of confidence in their data. Patients in the treatment group received adjuvant chemotherapy. No significant differences in GSH with the rate of local recurrence or death due to disease were found in any treatment group; however, one group receiving adjuvant therapy with metastases displayed significantly higher GSH levels [75].
14. Three metabolic clusters, MOC1–MOC3, were derived from multiomics data in the work of Sharma et al. [61] and validated using independent datasets. Patients with tissues characterized by the MOC2 profile had the lowest overall survival at >10 years, but MOC1 was lowest at 5 years. HRMAS analysis revealed that GSH, Asc, and Tau were significantly different between these clusters, but their relative levels have not been reported. Interestingly, these clusters showed a stronger association with long‐term (>10 years) survival than PAM50 subtypes.

#### 3.4.2. Tau Data in Relation to Prognosis/Response/Survival Was Reported in 14 Studies

##### 3.4.2.1. Tau Higher/Trended Higher in Poor Prognosis/Response/Survival in Two Studies

15. Tau levels are increased in more aggressive tumors in patients with poor prognosis [11].
16. Tau levels are higher in malignant tissues than in benign tissues [62].
17. In an HRMAS study, Tau levels tended to be higher (*p* = 0.12) in a subgroup with poor prognosis [63].

##### 3.4.2.2. Tau Higher/Trended Higher in Good Prognosis/Response/Survival in Nine Studies

18. The study presented above (8) showed higher levels of Tau in the GR group [60].
19. Tau tends to be higher in survivors after neoadjuvant chemotherapy with epirubicin and paclitaxel [64].
20. Tau levels were higher in survivors after neoadjuvant doxorubicin chemotherapy [65].
21. Giskeødegård et al. [66] studied a group of patients who had not undergone neoadjuvant therapy and found that Tau levels in surgical specimens were higher in patients who survived >5 years postsurgery compared to nonsurvivors. This difference was also observed in a subgroup of patients with ER positivity.
22. Tau increased with GPAM expression, and DHA decreased in tissues from patients with better overall survival [40].
23. Tumor tissues with higher levels of glutamate were correlated with improved overall survival. Tau and Cys levels were also found to be higher in these tissues [54].
24. HRMAS and in vivo magnetic resonance spectroscopy were used to determine whether metabolite profiles could differentiate indolent tumors from other tumors with favorable prognostic factors. Indolent tumors were defined as ER+, low‐grade, low Ki‐67 index, and negative lymph node metastases. Individual metabolite levels did not differ between the groups. However, tumors that did not recur had higher levels of Tau. Tau levels tended to be higher in patients with low‐grade ER+ tumors. Multivariate analysis predicted indolent tumors, tumors with recurrence, and lymph node metastases. However, Tau and other metabolite levels are elevated in tumors with lymph node metastases [67].
25. The study above (3) showed that Tau was higher in the MPS1 and MPS2 groups with better outcomes [15].
26. In the study of Debik et al. [68]. Tissue samples were obtained before treatment, 12 weeks into neoadjuvant therapy, and at surgical resection. Multivariate analyses at 12 weeks indicated that Tau and Asc levels predicted 5‐year survival. From the figure in this publication, Tau levels appear to be higher in patients with a favorable prognosis, although statistical data were NR.

##### 3.4.2.3. Tau Levels With Mixed Patient Outcome Results Were Reported in One Study

27. As reported above (6), the PA found mixed results when comparing the Tau/hypotaurine metabolic pathway in the four groups [34].

##### 3.4.2.4. Tau Levels Not Related to Prognosis/Response/Survival Response in One Study

28. There were no significant relationships between Tau levels in core needle biopsy tissue specimens in patients who experienced a pathologic complete response, partial response, or stable disease prior to neoadjuvant chemotherapy [69].

Of the 28 studies summarized, the majority found that GSH/GSSG levels were higher in tissues from patients with less favorable outcomes. Conversely, most studies found that Tau levels were higher in tissues from patients with more favorable outcomes.

### 3.5. Hormone Receptor Status

From the listed studies, most data have been collected for GSH and Tau in relation to hormone receptor status. However, the results varied. For GSH, two studies [27, 75] reported an increase in GSH in ER+ tumor tissue, whereas one study reported a decrease [42] and two other studies [10, 37] reported that GSH (and Cys) tended to lower levels, but these changes did not reach statistical significance. One study found elevated GSH levels in all tumors of patients fed a high‐carbohydrate diet, particularly in ER+ tissues [70]. Budczies et al. [55] did not detect any differences in GSH, Cys, Cys2, DHA, and Tau between ER+ and ER− tumor tissues. Similarly, no differences in GSH levels were found when comparing TNBC tissues to HR+/Her2− tissues [36].

For ER+ tumors, Tau levels were found to be higher in two studies [19, 30] and tended to be lower, but not significantly different, in another report [63], and two studies detected no difference [55, 90]. In PR+ tumor tissue, Tau was reported to be lower in one study [63] and higher in two others [19, 30]. In a subset of patient tumors with low Her2 expression, PA indicated that Tau/hypotaurine metabolism increased in HR− (ER−/PR−) tumors [43].

From these data, no conclusions can be reached regarding antioxidant levels and hormone receptor status.

### 3.6. Clinical Stage

DCIS is often referred to as stage 0 and has been included in this section. Few studies have compared antioxidant levels according to the clinical stage.

In a study comparing benign tissue to DCIS and IDC, GSH, GSSG, and Cys appeared higher in a heatmap figure, but no statistical analyses were performed [58]. However, in this study, PA indicated that Cys/methionine metabolism was significantly altered in IDC compared to other tissues.

Tau was found to be higher in the early stage [33] and higher along with Asc in DCIS than in DCIS with an invasive component (DCIS(IDC)) and invasive tumor tissue in one study [13]. Another study did not detect a significant difference between DCIS and DCIS(IDC) [71]. Neither GSH nor GSSG was related to stage in the targeted study [32].

No clear trend is observed between GSH or Tau in relation to clinical stage. That may be due to preponderance of stage II tumors in most of the studies.

### 3.7. Histologic Grade

Two studies reported that GSSG [15, 27] and one study showed that GSH [44] was increased in higher‐grade tumors. The latter study reported “homoglutathione” as one of the 12 most important metabolites differentiating low‐ from high‐grade tumors [44]. Although homoGSH has been reported in plant tissues, it has not been previously reported in breast cancer tissues and may be a misprint. Tau was reported to increase with tumor grade in three studies [16, 27, 54], but another [19] detected a trend toward higher levels in low‐grade tumors, but this was not statistically significant.

Based on these reports, GSH or GSSG levels increase with tumor grade, which is consistent with the patient outcome studies described above. In contrast, Tau was found to increase with grade in most studies, which is not consistent with the patient outcome studies above, indicating that lower Tau is associated with poor outcomes.

### 3.8. Subtypes

Tumor tissues are often characterized according to the genetically determined PAM50 subtypes. Three studies used cluster analysis to group tissues based on common metabolic traits and found that these clusters, which included many of the metabolites of interest, did not aligned with any of the PAM50 subtypes [11, 72, 75].

Metabolite data on subtypes are inconsistent, as few studies have made comparisons between similar tissues. The most widely metabolomics‐characterized subtypes are TNBC, LumA, B, and Her2, and are summarized below.

#### 3.8.1. TNBC

GSH, GSSG, Cys, CSSG [37] and GSH, Cys, and CG [42] were elevated in TNBC compared to ER+ tumor tissues. However, GSH, GSSG, Cys, and Cys2 levels were not significantly different between TNBC and ER+/PR+/Her2− tissue [36]. Additional studies have found that GSH, Tau, Asc, and Cys were not different between TNBC and LumA subtypes, which are ER+/PR+ [59]. A later study confirmed that Tau was not different between TNBC and LumA [12]. Asc was higher, but no difference was detected in GSH and Tau levels when comparing TNBC to DCIS, Her2+, LumA, and LumB subtypes [28]. Although multivariate analyses of metabolite profiles could distinguish TNBC from triple‐positive breast cancer (TPBC) and tissues based on Her2 status, Tau was NR to be significantly different in any univariate comparisons [90].

#### 3.8.2. Luminal

Two contrasting results, the first of which found that Tau was higher in LumA than in LumB [73], and the second determined that Tau is higher in LumB than in other subtypes [45]. Some of the conflicting evidence in metabolite data may be explained by the work of Yang et al. [23], who compared the spatial distribution of metabolites in LumA and Lum B tumors. Using punch biopsy specimens taken from the center and periphery of these tumors, they found that GSSG increased in the periphery of both subtypes compared to the center, whereas Tau levels were lower. In addition, there were spatial differences in the correlation between GSSG, Tau, and Asc and uptake of the PET tracer ^18^F‐fluorodeoxyglucose, with opposite trends detected for LumA and LumB. This illustrates the known heterogeneity of tumor tissue samples that confounds molecular analyses and supports the recent use of MSI‐based studies that can map this heterogeneity.

#### 3.8.3. Her2

Tau levels were higher in Her2+ than in Her2− tissues [63]. In a study of ER+ tumor tissues, Tau tended to be higher, but not statistically different, in Her2+ compared to Her2− [73]. However, a later study found that Tau was not related to HER2 status [19]. In a study comparing the expression of Her2−, Her2‐low, and Her2+ tissues, PAs determined that GSH, Cys, and Asc pathways did not correlate with Her2 expression [43]. However, Tau pathway metabolism increased with Her2 expression in HR tissues.

### 3.9. Antioxidant Markers in Proliferative Tissues

#### 3.9.1. Ki‐67

Only one report found that Asc, Cys, and CSSG were correlated with Ki‐67 staining [10]. In the immunohistochemical study, GSH did not correlate with Ki‐67 indices [75], and 2 HRMAS studies by the same group determined that Tau was not significantly different between Ki‐67 staining‐positive compared to ‐negative groups [19]. In a study comparing MRI parameters to immunohistochemical and metabolite levels, Tau was negatively correlated with the MRI diffusion coefficient only in Ki‐67‐negative tissues [74].

## 4. Conclusions

Although none of the reviewed metabolomics studies reported procedures to prevent sample oxidation, the finding that GSH or oxidized GSH levels are elevated in breast cancer compared to healthy or histologically normal tissues confirms earlier work and supports the continued use of these metabolomics technologies in antioxidant screening. An advantage of metabolomic approaches is the availability of data on other metabolites. These data also provide strong evidence that Tau and Asc levels are increased in breast tumor tissues. In addition, when comparing tumor tissue levels, it appears that higher GSH and lower Tau levels are indicators of poorer outcomes. The relationship between antioxidants and other tumor characteristics is less clear, but wider use of spatial metabolomics is warranted, as a single tissue can contain a mixture of tumor cells (e.g., DCIS, IDC, fibroblasts, and adipocytes), and methods such as MSI can metabolically characterize each cell type separately to remove the volume averaging effect of sample homogenization that occurs with many of the screening technologies. Overall, these data support the hypothesis that tumor tissue is commonly characterized by increased levels of metabolites contributing to a reduced intracellular environment, which can be useful as a prognostic factor and may be targeted for therapy.

## Conflicts of Interest

The author declares no conflicts of interest

## Funding

This work was supported the National Institutes of Health (Grant Number R01CA288969).

## Data Availability

Data sharing is not applicable to this article, as no new data were created or analyzed in this study.
